# Supplementary material for: The role of CPAP as a potential bridge to invasive ventilation and as a ceiling-of-care for patients hospitalized with Covid-19—An observational study
Source: PLoS One. 2020 Dec 31;15(12):e0244857. doi: 10.1371/journal.pone.0244857 (PMC7774971; doi:10.1371/journal.pone.0244857)

**Supplementary Figure 1: Conceptual framework of relationship between exposure and confounding variables and mortality**


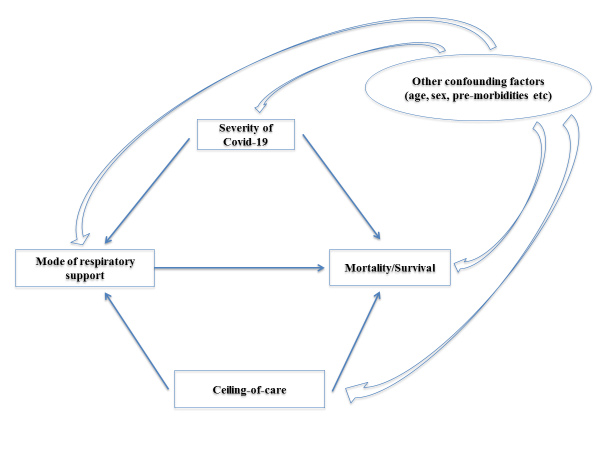

Supplement: S1 Fig — (DOCX) [file pone.0244857.s001.docx]
