# Supplementary material for: The role of CPAP as a potential bridge to invasive ventilation and as a ceiling-of-care for patients hospitalized with Covid-19—An observational study
Source: PLoS One. 2020 Dec 31;15(12):e0244857. doi: 10.1371/journal.pone.0244857 (PMC7774971; doi:10.1371/journal.pone.0244857)
Supplement: S2 Fig — (DOCX) [file pone.0244857.s002.docx]

**Supplementary Figure-2 Flow diagram of patients included in the study and mortality by mode of respiratory support and ceiling-of-care**
